# The Antileishmanial Potential of C-3 Functionalized Isobenzofuranones against *Leishmania (Leishmania) Infantum Chagasi*

**DOI:** 10.3390/molecules201219857

**Published:** 2015-12-14

**Authors:** Wagner Luiz Pereira, Raphael de Souza Vasconcellos, Christiane Mariotini-Moura, Rodrigo Saar Gomes, Rafaela de Cássia Firmino, Adalberto Manoel da Silva, Abelardo Silva Júnior, Gustavo Costa Bressan, Márcia Rogéria Almeida, Luís Carlos Crocco Afonso, Róbson Ricardo Teixeira, Juliana Lopes Rangel Fietto

**Affiliations:** 1 Departamento de Química, Universidade Federal de Viçosa, Av. P.H. Rolfs, S/N, Viçosa, MG, 36.570-900, Brazil; wagner.pereira@ufv.br (W.L.P.); adalbert31@hotmail.com (A.M.S.); 2 Departamento de Biologia Geral, Universidade Federal de Viçosa, Av. P.H. Rolfs, S/N, Viçosa, MG, 36.570-900, Brazil; raphael.biomed@gmail.com (R.S.V.); chrismariotini@yahoo.com.br (C.M.-M.); rafaelacfirmino@gmail.com (R.C.F.); 3 Instituto Nacional de Biotecnologia Estrutural e Química Medicinal em Doenças Infecciosas (INBEQMeDi), Instituto de Física de São Carlos, Av. Trabalhador São Carlense, 400, Caixa Postal 369, São Carlos, SP, 13.560-970, Brazil; 4 Departamento de Ciências Biológicas, Instituto de Ciências Exatas e Biológicas—ICEB/NUPEB, Campus do Morro do Cruzeiro, Universidade Federal de Ouro Preto, Ouro Preto, MG, 35.400-000, Brazil; rodrigosaar@nupeb.ufop.br (R.S.G.); afonso@nupeb.ufop.br (L.C.C.A.); 5 Departamento de Veterinária, Universidade Federal de Viçosa, Av. P.H. Rolfs, S/N, Viçosa, MG, 36.570-900, Brazil; aberlardo.junior@ufv.br; 6 Departamento de Bioquímica e Biologia Molecular, Av. P.H. Rolfs, S/N, Viçosa, MG, 36.570-900, Brazil; gustavo.bressan@ufv.br (G.C.B.); marcia@ufv.br (M.R.A.)

**Keywords:** *Leishmania (L.) infantum chagasi*, visceral leishmaniasis, isobenzofuranones, phthalides, *in vitro* leishmanicidal activity

## Abstract

Leishmaniases are diseases caused by protozoan parasites of the genus *Leishmania*. Clinically, leishmaniases range from cutaneous to visceral forms, with estimated global incidences of 1.2 and 0.4 million cases per year, respectively. The treatment of these diseases relies on multiple parenteral injections with pentavalent antimonials or amphotericin B. However, these pharmaceuticals are either too toxic or expensive for routine use in developing countries. These facts call for safer, cheaper, and more effective new antileishmanial drugs. In this investigation, we describe the results of the assessment of the activities of a series of isobenzofuran-1(3*H*)-ones (phtalides) against *Leishmania (Leishmania) infantum chagasi,* which is the main causative agent of visceral leishmaniasis in the New World. The compounds were tested at concentrations of 100, 75, 50, 25 and 6.25 µM over 24, 48, and 72 h. After 48 h of treatment at the 100 µM concentration, compounds **7** and **8** decreased parasite viability to 4% and 6%, respectively. The concentration that gives half-maximal responses (LC_50_) for the antileishmanial activities of compounds **7** and **8** against promastigotes after 24 h were 60.48 and 65.93 µM, respectively. Additionally, compounds **7** and **8** significantly reduced parasite infection in macrophages.

## 1. Introduction

The leishmaniases are parasitic diseases that are caused by protozoa of the genus *Leishmania*. These parasites belong to the order Kinetoplastida and family Trypanosomatidae, which includes species of obligatory intracellular protozoan parasites. Common hosts include rodents, canines and primates, including humans [1,2]. *Leishmania* infections can present with different clinical manifestations depending on the parasite species and the host-parasite relationship. Visceral leishmaniasis (VL) is a progressive and frequently fatal disease caused by *Leishmania (Leishmania) infantum* (synonymous with *Leishmania (Leishmania) infantum chagasi*) [3]. VL is one of the most neglected diseases in the world and affects millions of people worldwide [4]. Approximately 500,000 new cases occur annually, and 90% of all VL cases occurring in India, Bangladesh, Sudan, South Sudan, Ethiopia and Brazil [4].

Currently, several investigations are being conducted to search for alternative treatments for leishmaniases [5] because of the small number of available drugs and the development of resistance or decreased sensitivity of parasite strains to existing treatments that are utilized for human therapy [5]. These studies have sought new methods and targets for diagnosis, new vaccine candidates and new rationally designed drugs that can be applied not only in humans, but also in dogs because canines are considered the major reservoirs of several species of *Leishmania* in the home environment and outdoors [6,7].

Historically, chemotherapy for leishmaniases has relied on the use of pentavalent antimonial drugs, such as *N*-methylglucamine antimoniate, which is one of the most widely used drugs [8]. However, the use of this drug has limited clinical potential due to the occurrence of serious side effects and high incidence of disease recurrence [9]. Pentamidine is another antileishmanial agent, but it is inadequate as a first-line treatment because of its high toxicity [10]. Amphotericin B has been used as a second-choice drug in the treatment of leishmaniasis since the 1960s, but the rate of resistance against this drug is high [10]. Two other examples of antileishmanial compounds are miltelfosine and paromomocyn. The major limitation of miltefosine is teratogenicity and this precludes its use in women of child-bearing age [11,12]. The most common side effect associated with paromomycin is the ototoxicity, as well as liver function problems. In patients treated with the ointment formulation skin rashes, local pruritus and burs have been the most common side effects encountered [13] Additionally, the drugs that are available for leishmaniasis chemotherapy are, in general, expensive. Another alternative is combination therapy, which has been used to decrease the duration and price of treatment and parasite resistance [14]. The aforementioned problems illustrate a pressing need to develop new antileishmanial drugs.

**Figure 1:**
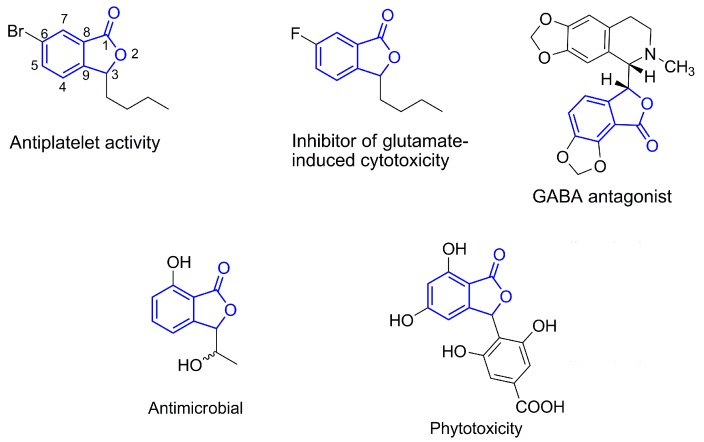
Example isobenzofuran-1(3*H*)-ones and their biological activities.

Compounds containing a benzene ring fused to a γ-lactone ring are termed isobenzofuran-1(3*H*)-ones (also known as phthalides). This unit is present in the structure of several natural products [15,16]. In particular, isobenzofuran-1(3*H*)-ones functionalized at the C-3 position stand out for their biological activities, which include antiplatelet [17], GABA receptor antagonist [18], glutamate-induced cytotoxicity inhibitor [19], phytotoxicity [20], cytotoxicity [21,22] and antimicrobial activity [23]. Examples of isobenzofuranones and their associated bioactivities are shown in Figure 1, where the isobenzofuran-1(3*H*)-one unit is highlighted in blue.

Considering their various biological activities and our research interest in isobenzofuran-1(3*H*)-ones [22,24], we herein describe the results of our investigation of the antileishmanial activities of C-3 functionalized isobenzofuranones on *Leishmania (Leishmania) infantum chagasi*.

## 2. Results and Discussion

**Scheme 1:**
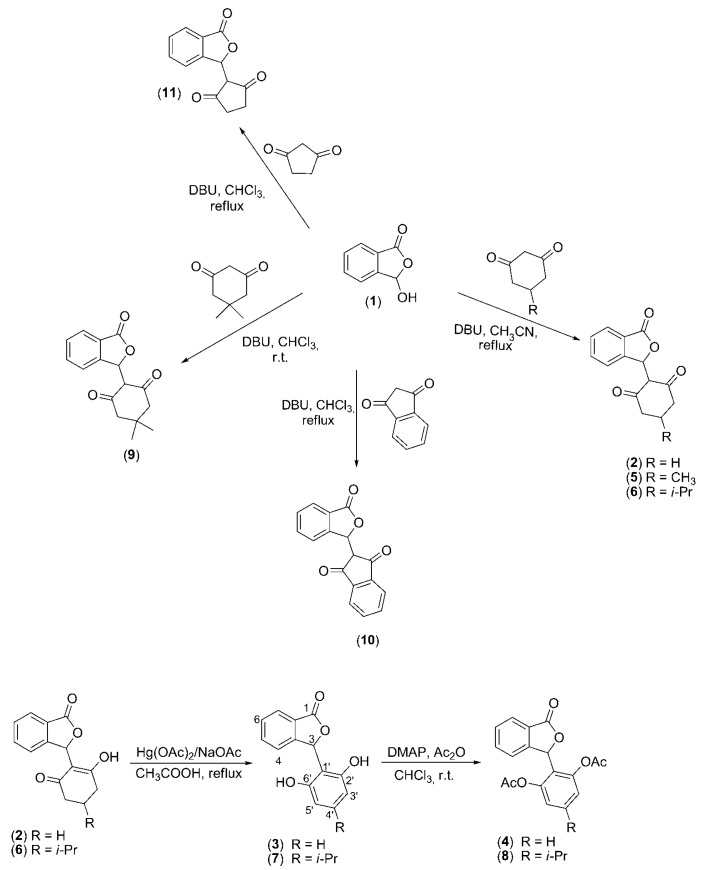
Syntheses of isobenzofuranones **2**–**11**.

It is well known that amphotericin B, which is used for the treatment of visceral leishmaniasis, is an effective antibiotic that also possesses antifungal activity [10]. Because previous studies have demonstrated that isobenzofuranones display antifungal activities [25,26], we hypothesized that isobenzofuranones **2**–**11** (Scheme 1) would also exhibit antileishmanial activities.

Thus, we evaluated the antileishmanial activities of isobenzofuranones **2**–**5** and **7**–**11** (Scheme 1) against the pathogenic agent of visceral leishmaniasis *Leishmania (L.) infantum chagasi* (*syn. Leishmania infantum*). Because of limited amount of compound **6** available during the progress of the biological assays, this compound was not evaluated. The first approach involved evaluations of the direct actions of these compounds on the promastigote form of the parasite.

**Figure 2:**
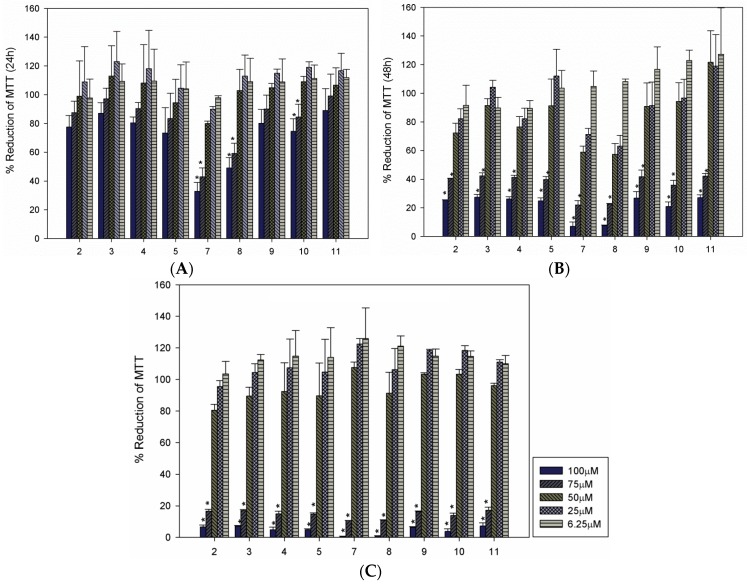
Leishmanicidal activities of isobenzofuranones **2**–**5** and **7**–**11** against promastigotes. MTT viability assays were used to evaluate the promastigotes of *L. infantum* after 24 (**A**); 48 (**B**); and 72 h (**C**) of treatment. The results are presented as the averages and the SDs of three independent experiments that were performed in triplicate. The * indicates significant differences compared with the control at *p* < 0.05.

We observed that all of the compounds were toxic to the parasite (Figure 2). After 24 h of treatment, the 100 μM concentrations of compounds **7** and **8** significantly reduced parasite viability by over 50% (Figure 2A). After 48 and 72 h, all of the isobenzofuranones were effective in decreasing parasite viability (Figure 2B,C). Additionally, 100% parasite death was observed after 72 h of treatment with compounds **7** and **8** (Figure 2C). Table 1 illustrates the LC_50_ antileishmanial activity values of compounds **2**–**5** and **7**–**11** against the promastigote form of *L. (L.) infantum (syn. L. chagasi*). The most active compounds, *i.e.*, **7** and **8**, exhibited LC_50_ values between 60.48–59.75 and 65.93–58.46 µM, respectively.

Thus, the greatest antileishmanial efficacies against the promastigotes were observed for isobenzofuranones **7** and **8**; importantly, these compounds share the common feature of an aromatic ring that is directly attached at the C-3 position of the isobenzofuranone nucleus. Moreover, this aromatic ring is oxygenated at the C-2′/C-6′ positions and exhibits an alkyl group (isopropyl) attached to C-4′ (see Scheme 1 for numbering). Thus, these structural features seem to be important in terms of the antileishmanial activities of the investigated compounds. Notably, a previous investigation of the cytotoxicities of compounds **2**–**11** against the K562 and U937 cancer cell lines [22] revealed that the most active compounds exhibited structural features similar to these aforementioned features. Indeed, isobenzofuranone **7** was one of the most active compounds against K562 exhibiting an LC_50_ of 2.79 μM and was even more potent than the anticancer agent etoposide (VP-16), which was used as a positive control. Due to the superior antileishmanial activities of compounds **7** and **8** after 24 h (Figure 2A), these compounds were selected for further evaluation.

**Table 1:** Antileishmanial efficacies of isobenzofuranones against promastigotes.

Compound	LC_50_ (µM) ^a^		
	24 h	48 h	72 h
**2**	74.54	65.55	58.96
**3**	>100	65.73	59.08
**4**	>100	65.69	59.20
**5**	>100	61.69	58.96
**7**	60.48	62.72	59.75
**8**	65.93	63.90	58.46
**9**	75.01	54.78	60.51
**10**	74.27	61.84	60.53
**11**	>100	30.79	60.17

^a^ The LC_50_ values were calculated at 24, 48 and 72 h of treatment. The data are representative of three independent experiments.

The second investigation examined the toxicities isobenzofuranones **7** and **8** against macrophages, which are the main cells that are infected by *Leishmania* parasites in leishmaniasis diseases. We observed low levels of toxicity at high isobenzofuranones concentrations (100 µM) and selected a lower dose to perform the infection assays (75 µM). Both compounds decreased the levels of infection and reduced the numbers of intracellular parasites per infected cell. Together, these results suggest that these compounds may affect both infection capability and intracellular proliferation. In an investigation of the possible mechanism of action of C-3 functionalized isobenzofuranones against the HL-60 cancer cell line, Logrado and collaborators found that the active compounds exerted their effect on HL-60 cells via direct DNA damage or the generation of free radicals [21]. Although the mechanisms of action of the most active compounds were not investigated here, it is possible that the antileishmanial activities that were observed for compounds **7** and **8** are also related to these mechanisms. Conceivably, due to the intense replication of this parasite during acute infection, it could be strongly affected by DNA damage or free radicals. Indeed, reports in the literature have indicated that anti-cancer drugs that affect cellular proliferation can be used as anti-microbial agents because both cell types share common behaviors concerning their high proliferative capabilities [27,28,29]. Indeed, a recent work evidenced the antileishmanial actions of other two isobenzofuranone derivatives and indicated that these actions were due to the induction of reactive oxygen species (ROS)-mediated apoptosis-like cell death and the inhibition of topoisomerases [30].

**Figure 3:**
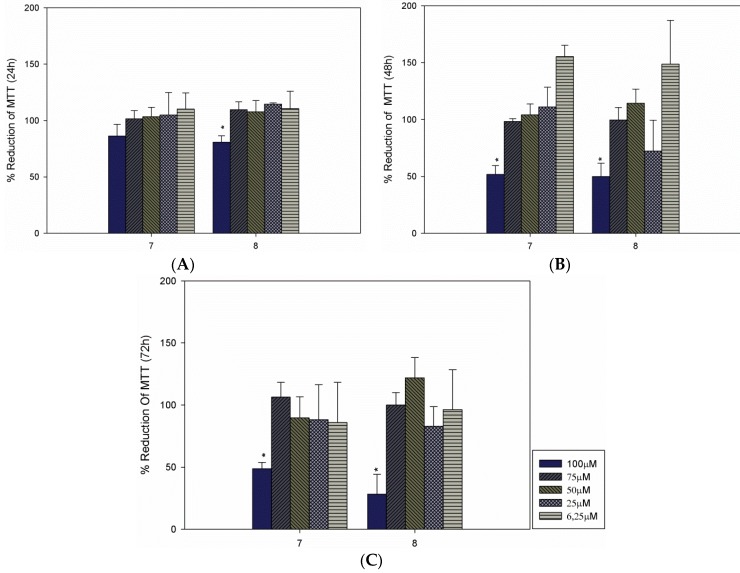
Toxicities of isobenzofuranones **7** and **8** to RAW macrophages. MTT viability assays were performed after 24 h (**A**); 48 h (**B**) and 72 h (**C**) of treatment. The results are presented as the averages and the SDs of three independent experiments that were performed in triplicate. The * indicates significant differences compared with the control at *p* < 0.05.

To assess whether the compounds tested on the parasites affected cell viability, RAW macrophages were maintained for 24, 48 and 72 h in the presence of compounds **7** and **8** at concentrations that ranged from 6.25 to 100 µM. There were no significant differences in cellular viability after 24 h with the tested concentrations of compound **7** (Figure 3A). However, the viabilities of the macrophages were significantly affected after 48 and 72 h of treatment at 100 µM (Figure 3B,C). Regarding compound **8**, the 100 µM concentration affected the viability after 24 h (Figure 3A). However, concentrations of 75 µM and lower did not seem to affect the macrophages (Figure 3). Based on these results, the 75 µM concentration was selected for the infection assays.

**Figure 4:**
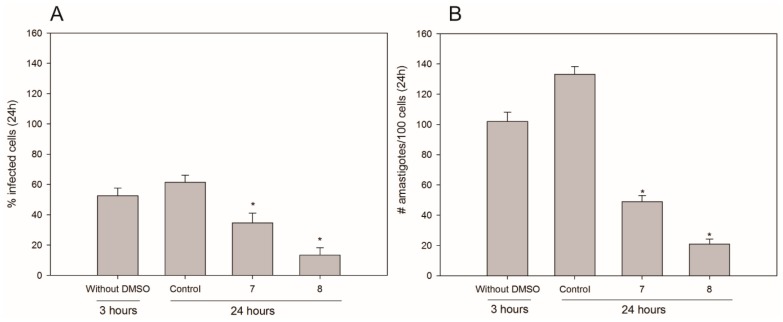
Leishmanicidal activities of compounds **7** and **8** in infected RAW macrophages. The percentages of infected macrophages (**A**); and the numbers of intracellular amastigotes per 100 cells (**B**) were assessed. The first column (without DMSO) is the control after 3 h of infection and before the addition of 1% DMSO. The second column is the sample assay after 24 h of infection in the presence of DMSO. The last two columns are the samples with isobenzofuranones **7** and **8** at the concentration of 75 µM in 1% DMSO. The data are presented the averages and the SDs of three independent experiments that were performed in triplicate. The * indicates significant differences compared with the control at *p* < 0.05.

Next, macrophages were infected with *L. (L.) infantum chagasi* and subsequently treated with isobenzofuranones **7** and **8** at 75 µM for 24 h. Regarding compound **7**, a reduction of the number of infected cells by approx. 65% was observed (Figure 4A). This effect was even more pronounced for compound **8**, which reduced the number of infected cells by 76% (Figure 4B).

**Figure 5:**
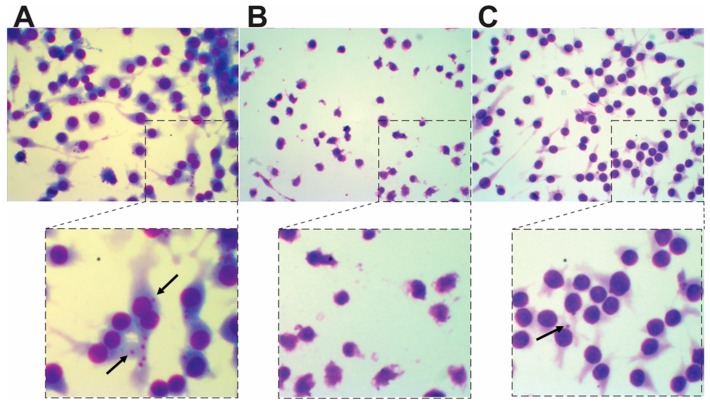
Representative micrographs showing RAW macrophages infected with *L. (L) infantum chagasi* that were or were not treated compounds **7** or **8**. The infected macrophages were treated for 24 h with 1% DMSO (control) (**A**); 75 µM of compound **7** (**B**); or 75 µM of compound **8** (**C**). The pictures are representative of all of the experiments, which were conducted as independent triplicates. The upper pictures are magnified at 100x, and the lower panels are zoomed in images of the selected parts of the upper pictures. The black arrows indicate intracellular amastigotes.

Importantly, although compound **7** did not affect the viability of the macrophages as assessed with the MTT method, we observed visible changes in their morphologies (Figure 5B). This phenotype was not observed for compound **8**, which did not cause any visible changes in the overall cell morphology (Figure 5C). These findings suggest that the compounds **7** and **8** differ in their mechanism of action, which should be better investigated in future studies.

## 3. Experimental Section

### 3.1. Syntheses

The syntheses of the isobenzofuranones were performed as previously reported [22]). Compounds **2**–**11** were prepared using condensation, aromatization and acetylation reactions (Scheme 1). Briefly, DBU-mediated condensation reactions between commercially available phthalaldehydic acid (**1**) and cyclic and acyclic 1,3-diketones produced compounds **2**, **5**, **6**, **9**, **10** and **11**. The aromatization of phthalides **2** and **6** produced the aromatic derivatives **3** and **7**. The acetylation of compounds **3** and **7** led to the preparation of substances **4** and **8**. The structural characterizations of these compounds have been described previously [19]. Stock solutions of the compounds were prepared by dissolving them in 100% DMSO. The solutions were maintained at 4 °C until use. Due to the limited amount of compound **6**, this phtalide was not evaluated.

### 3.2. Obtaining of Promastigotes Parasites and Leishmanicidal Activity Evaluation

*L. (L.) infantum chagasi* strain M2682 was grown in Grace’s insect medium supplemented with 10% heat-inactivated fetal calf serum, 2 mM L-glutamine and 100 U/mL penicillin G potassium at pH 6.5 and 26 °C. On the third day (exponential phase), the promastigote parasites were washed with culture medium, plated at 1 × 10^6^ *Leishmania*/mL without (control) or with 100 μM of isobenzofuran-1(3*H*)-ones **2**–**5** and **7**–**11**, and incubated at 26 °C for 24, 48 and 72 h. After incubation, 1 mM of 3-(4,5-dimethylthiazol-2-yl)-2,5-diphenyltetrazolium bromide (MTT) was added and followed by an incubation for 2 h at 26 °C. Subsequently, the parasites were centrifuged at 1400× *g*, the supernatants were discarded, and 100 μL of dimethylsulfoxide (DMSO) was added to the pellet, which was subsequently stirred for 15 min and reading at 595 nm. The calculations of the viable parasites were performed using the following equation:

$$\frac{absorbance\;of\;the\;test-absorbance\;of\;the\;plate}{absorbance\;of\;the\;control-absorbance\;of\;the\;plate}\times 100$$

### 3.3. Cultivation of Macrophages and Cytotoxicity Evaluation

The mouse leukemic macrophage cell line RAW (RAW) were grown in Roswell Park Memorial Institute Media (RPMI) supplemented with 10% (*v*/*v*) heat-inactivated fetal calf serum (LGC Bio^®^, Petaluma, CA, USA), 100 U/mL penicillin (Sigma^®^, St. Louis, MO, USA), and 100 μg/mL streptomycin (Sigma^®^) in flasks of 75 or 150 or 300 cm^3^, which were maintained at 37 °C in an atmosphere with 95% humidity and 5% CO_2_. Thereafter, the macrophages were transferred to 96-well plates at a concentration of 1 × 10^6^ macrophages/mL. The compounds were added to the culture medium at different concentrations and incubated at 37 °C (5% CO_2_) for 24, 48 and 72 h. After incubation, 1 mM of MTT was added in each well, and the cultures were returned to the incubator for 2 h. Next, the culture medium was discarded, and 100 μL DMSO was added, and the wells were stirred for 15 min and subsequently read at 595 nm. The calculations of the percentages of viable cells were performed as described in the previous section.

### 3.4. Infection Assays

The macrophages were left to adhere in the 24-well plate (which included a sterile coverslip for each well) for 90–120 min at a concentration of 1 × 10^6^/mL. The cells were washed twice with PBS to remove the non-adhered cells. For infection, 3–5 × 10^6^ parasites/mL suspended in RPMI with 10% fetal calf serum were added to macrophage cells, which were then left to be infected for 3 h (5 *Leishmania* per macrophage). Next, the wells were washed twice with PBS to remove the parasites that did not infect macrophages. Subsequently, RPMI with 10% fetal calf serum containing the compounds at the different concentrations specified in the figures was added. All samples, including the controls, contained 1% DMSO (final DMSO concentration in the working solution). The cultures were incubated for 24 or 48 h in the same conditions described above. After incubation, the cells were fixed on coverslips with methanol and stained with Fast Panoptic FAST kit (Panreac AppliChem, Darmstadt, Germany) according to the manufacturer’s recommendations. All assays were performed in triplicate, and the stained coverslips were analyzed by light microscopy. We evaluated the numbers of infected macrophages and the numbers of intracellular amastigotes per 100 macrophages.

## 4. Conclusions

The potential of diverse C-3 substituted isobenzofuran-1(*3H*)-ones as antileishmanial agents against *Leishmania (L.) infantum chagasi (syn. Leishmania infantum*) was evaluated. The efficacies of the compounds against promastigote viability were dependent on the type of substituent attached to the C-3 position. Among the compounds evaluated, the isobenzofuranones **7** and **8** were found to be capable of significantly reducing the percentage of infected macrophages and the number of intracellular amastigotes per infected macrophage. Based on the results obtained, these isobenzofuranones may represent a novel scaffold that can be exploited to develop of new drugs for the treatment of leishmaniasis.
